# Comprehensive Review of *Tolypocladium* and Description of a Novel Lineage from Southwest China

**DOI:** 10.3390/pathogens10111389

**Published:** 2021-10-27

**Authors:** Feng-Ming Yu, Kandawatte Wedaralalage Thilini Chethana, De-Ping Wei, Jian-Wei Liu, Qi Zhao, Song-Ming Tang, Lu Li, Kevin David Hyde

**Affiliations:** 1 Center of Excellence in Fungal Research, Mae Fah Luang University, Chiang Rai 57100, Thailand; 6171105508@lamduan.mfu.ac.th (F.-M.Y.); kandawatte.thi@mfu.ac.th (K.W.T.C.); wei_deping@cmu.ac.th (D.-P.W.); liujianwei@mail.kib.ac.cn (J.-W.L.); 6171105516@lamduan.mfu.ac.th (S.-M.T.); 2 School of Science, Mae Fah Luang University, Chiang Rai 57100, Thailand; 3 Key Laboratory for Plant Diversity and Biogeography of East Asia, Kunming Institute of Botany, Chinese Academy of Sciences, Kunming 650201, China; zhaoqi@mail.kib.ac.cn (Q.Z.); lilu@mail.kib.ac.cn (L.L.); 4 The Germplasm Bank of Wild Species, Yunnan Key Laboratory for Fungal Diversity and Green Development, Kunming Institute of Botany, Chinese Academy of Sciences, Kunming 650201, China; 5 Yunnan Key Laboratory for Fungal Diversity and Green Development, Kunming 650201, China; 6 Institute of Applied Fungi, Southwest Forestry University, Kunming 650224, China; 7 School of Chemical Engineering, Guizhou Institute of Technology, Guiyang 550003, China; 8 Biotechnology and Germplasm Resources Institute, Yunnan Academy of Agricultural Science, Kunming 650205, China

**Keywords:** new taxon, diversity, ecology, host shift, multi-gene, mycoparasite, taxonomic key

## Abstract

*Tolypocladium*, a diverse genus of fungicolous fungi belonging to *Ophiocordycipitaceae*, includes saprotrophic soil inhabitants, plant endophytes and pathogens of insects, nematodes, rotifers, and parasites of truffle-like fungi. Here, we review the research progress achieved for *Tolypocladium* regarding its taxonomy, species diversity, geographic distribution, host affiliations and ecological diversity. Furthermore, an undescribed taxon from China was established using morphology and multi-gene phylogeny. *Tolypocladium inusitaticapitatum* is introduced as a new species parasitizing ectomycorrhizal *Elaphomyces* species. It is diagnosed by its irregularly enlarged fertile heads and lemon, yellow-to-dark-brown, smooth and nearly cylindrical stipe. Phylogenetic analyses based on SSU, LSU, ITS, *TEF*1-*α* and *RPB*2 sequence data showed *T. inusitaticapitatum* to be an independent lineage separated from *T. flavonigrum* in the clade comprising *T. capitatum*, *T. fractum* and *T. longisegmentatum*. A key for identifying the sexual *Tolypocladium* species is also provided.

## 1. Introduction

Fungal species establish antagonistic to mutualistic associations with numerous prokaryotes and eukaryotes, including bacteria, algae, animals, plants and other fungi [1]. More than 1500 fungicolous taxa are widely distributed in aquatic and terrestrial ecosystems from tropical to polar regions [1]. Their hosts are ecologically diverse across the fungal kingdom. Truffle-like fungi are hypogeous and taxonomically distributed in Ascomycota and Basidiomycota [2]. Some truffle-like fungi were reported to be hosts of fungicolous species belonging to *Absidia* Tiegh., *Battarrina* (Sacc.) Clem. and Shear, *Entoloma* P. Kumm., *Hypocrea* Fr., *Hypomyces* (Fr.) Tul. and C. Tul., *Hypoxylon* Bull., *Melanospora* Corda, *Sporothrix* Hektoen and C.F. Perkins, and *Tolypocladium* W. Gam [1,3].

*Tolypocladium* W. Gams was established based on three soil-inhabiting asexual species: *Tolypocladium cylindrosporum* W. Gams, *T. geodes* W. Gams and *T. inflatum* W. Gams (the type species) [4]. Hodge and colleagues linked the asexual *T. inflatum* to the sexual species *Cordyceps subsessilis* Petch [5]. Subsequently, Sung and colleagues introduced the sexual genus *Elaphocordyceps* G.H. Sung and Spatafora and linked it to the asexual *Tolypocladium* and some species within *Verticillium* Nees based on multigene phylogeny [6]. Moreover, Sung and colleagues transferred the species of *Cordyceps sensu lato* that parasitize ectomycorrhizal *Elaphomyces* (18 species and two forma), cicada nymphs (*C. inegoënsis* Kobayasi, *C. paradoxa* Kobayasi, and *C. toriharamontana* Kobayasi) and beetle larvae (*C. subsessilis*) to *Elaphocordyceps* [6]. *Chaunopycnis* was established by Gams to accommodate *Ch. alba*, which resembles *Tolypocladium* in conidiogenesis [7]. Later, Quandt and colleagues synonymized *Chaunopycnis* and *Elaphocordyceps* under *Tolypocladium*, following the “One Fungus One Name” rule, as *Tolypocladium* is much more widely known, medicinally important and an older genus [4,6,7,8].

Most *Tolypocladium* species are *Elaphomyces*-attacking mycoparasites, except for few entomopathogens [9,10]. The evolution of host specificity and the dynamics of host jumping were investigated by several researchers using molecular data [6,8,11,12,13,14,15]. Nikoh and Fukatsu inferred that there was a shift from entomoparasitism to mycoparasitism during the evolution of the *Cordyceps*-like fungi [11]. However, with the addition of more gene regions and taxa, insect pathogens such as *T. paradoxum* and *T. inflatum* were found to be clustered with some parasites on truffles. The researchers explained that the ancestral ecology was a truffle parasitism, with multiple switches to insect pathogenicity [6,8,12]. Notably, the interspecific relationships of closely related *Tolypocladium* species are weakly supported and inconsistently resolved with different datasets [6,8,13,14]. To compensate for the shortage of limited loci, Quandt and colleagues performed genome-scale phylogenetic analyses based on two entomopathogens (*T. ophioglossoides* and *T. capitatum*) and two mycoparasites (*T. inflatum* and *T. paradoxum*) and demonstrated that truffle parasites form a monophyletic clade. They suggest that this lineage is derived as a result of a single ecological transition or host-jumping from insects to fungi [15].

A successful infection caused by fungal pathogens generally undergoes host recognition, attachment, and then infection and degradation, depending on the gene content, expression, or regulation [16]. *Tolypocladium* is recognized as an ideal candidate for investigating the mechanisms associated with host-jumping [15,16]. Quandt and colleagues researched the set of genes that are differentially regulated in *Tolypocladium* species during their first encounter with their hosts [16]. They found that PTH11-related G-protein-coupled receptors (GPCRs), predicted to be involved in host recognition, were up-regulated in *T. ophioglossoides* when grown on media containing insect cuticles [16]. Furthermore, a divergent chitinase and an adhesin gene, *Mad*1, were significantly up-regulated on media containing *Elaphomyces* [16]. According to the transcriptomic data, genes involved in redox reactions and transmembrane transport were the most overrepresented during *T. ophioglossoides* growth on *Elaphomyces* media. However, the genes involved in secondary metabolism may not be necessary for the parasitism of truffles as their products are only highly expressed during the growth on insect tissues [16].

To date, *Tolypocladium* comprises 41 species (Table 1) with a cosmopolitan distribution [2,17]. Some of them produce various secondary metabolites, such as cyclosporin, efrapeptins, ophiocordin and ophiosetin [18]. They have been widely used in biopharmaceuticals and biocontrol [18]. During an investigation of fungi in Yunnan Province, Southwest China, an undescribed *Tolypocladium* species was discovered on *Elaphomyces* sp. The present study aimed to (i) systematically review species diversity, hosts/habitat, geographical distribution and host affiliations of *Tolypocladium* species, (ii) broaden the knowledge of species diversity and host shifts in *Tolypocladium* species, (iii) refine the diagnostic characters of the interspecific classification of *Tolypocladium* in sexual morphs and provide a taxonomic key.

## 2. Results

### 2.1. Phylogenetic Placement

**Figure 1:**
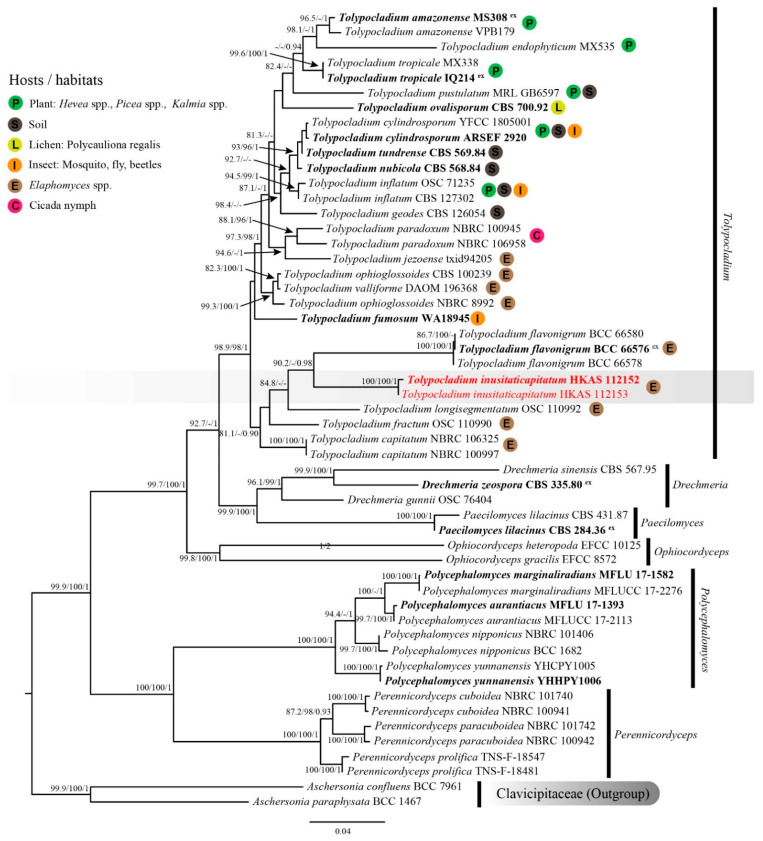
Maximum likelihood (ML) tree of *Tolypocladium inusitaticapitatum* and its allies within *Ophiocordycipitaceae* inferred from combined SSU, LSU, ITS, *TEF*1-*α* and *RPB*2 dataset. Bootstrap support values for ML ≥ 80 of SH-aLRT or 95 of UFB and posterior probability for BI ≥ 0.90 are indicated above the nodes and separated by ‘-/-/-’ (SH-aLRT/UFB/BIPP). Specimens of the current study are given in red. Type specimens are in bold and the superscript ‘ex’ indicates ex-type.

The combined SSU, LSU, ITS, *TEF*1-*α* and *RPB*2 sequence dataset comprised 35 species, containing 5384 nt (SSU: 1–1536, LSU: 1537–2441, ITS: 2442–3306, *TEF*1-*α*: 3307–4264, *RPB*2: 4265–5384) after the alignment (including gaps). Among them, 3731 bp (base pairs) were conserved, 378 variable, parsimony-uninformative, and 1275 parsimony-informative. The ML and BI analyses resulted in phylogenetic trees with a similar topology. The ML tree with a final log-likelihood of −27186.604 is shown in Figure 1. Specimens HKAS 112152 and HKAS 112153 clustered together and formed a distinct clade with strong support values (SH-aLRT = 100, UFB = 100 and BIPP = 1), indicating a conspecific relationship. These two specimens separated from other *Tolypocladium* species with SH-aLRT = 90.2 and BIPP = 0.98 support values. However, their LSU sequences showed an 11 bp difference (1.28%) across the 862 bp region, contributing to the different branch lengths in the phylogenetic tree. Based on the available molecular data for *Tolypocladium* species, some differences are known to occur due to intraspecific variations in the LSU sequences, ranging from 0.25 to 1.28% (Table 2).

Specimens *Tolypocladium inusitaticapitatum* (China), together with four *Tolypocladium* species occurring on *Elaphomyces* spp., i.e., *T. capitatum* (intercontinental distribution), *T. flavonigrum* (Thailand), *T. fractum* (USA) and *T. longisegmentatum* (intercontinental distribution), formed a monophyletic clade with weak support (SH-aLRT = 81.1, UFB = 82 and BIPP = 0.90. UFB values not shown in the ML tree). *Tolypocladium inusitaticapitatum* formed a separate clade sister to *T. flavonigrum*. However, the nucleotide comparison between *T. inusitaticapitatum* (holotype: HKAS 112152) and *T. flavonigrum* (holotype: BCC 66576) showed 154 bp (26.78%) differences across 575 bp ITS, 87 bp (9.83%) differences across 885 bp LSU, and 47 bp (4.99%) differences across 942 bp *TEF*1-*α* (including gaps), respectively. The phylogenetic evidence suggested that these two specimens represent new species.

### 2.2. Taxonomy

*Tolypocladium* W. Gams, Persoonia 6: 185 (1971); emended by Quandt and colleagues, IMA Fungus 5: 125 (2014).

Index Fungorum number: IF10242; Facesoffungi number: FoF 10425.

*Synonyms*: *Chaunopycnis* W. Gams, Persoonia 11: 75 (1980).

*Elaphocordyceps* G.H. Sung and Spatafora, Stud. Mycol. 57: 36 (2007).

*Type species*: *Tolypocladium inflatum* W. Gams 1971.

*Morphological characterization*: *Sexual morph*: *Stromata* arise directly from the host and are sometimes indirectly connected to the host through rhizomorph-like structures. They range from solitary to several and can be simple or branched. *Stipe* is fibrous to tough, rarely fleshy, dark-brownish to greenish with an olivaceous tint, rarely whitish, cylindrical and enlarges near the fertile part. The *fertile part* is clavate- to capitate-shaped and varies in color. *Perithecia* are partially to completely immersed, or superficial, or produced on a highly reduced stromatic pad, and ostiolate. *Asci* are unitunicate and long cylindrical with a thickened apical cap. *Ascospores* are filiform, approximately as long as asci, multi-septate, typically disarticulate into part-spores, and are occasionally non-disarticulating when mature (e.g., *T. ramosum*). *Part-spores* are hyaline, fusiform to cylindrical with round to truncate ends [6,8]. *Asexual morph*: They are *Tolypocladium*-, *Chaunopycnis*-, or *Verticillium*-like. *Colonies* are white, cottony and grow slowly on artificial media (e.g., potato dextrose agar, Czapek–Dox agar, malt extract agar, Sabouraud Glucose agar and water agar). *Conidiophores* usually are short and bear lateral or terminal phialides whorls. *Phialides* usually are swollen at the base and thin, often with bent necks. *Conidia* are globose to oval, one-celled, hyaline, smooth, and aggregative in small heads at the tips of the phialides [4,23].

*Hosts and habits*: Found in terrestrial and humid environments. Species of *Tolypocladium* parasitize hypogeous *Elaphomyces* (20 species including the novel species described in this study), cicada nymphs (4 species), beetle larvae (*T. inflatum*), pupa of the bagworm moth (*T. fumosum*), mosquito larvae (*T. extinguens*), and even bdelloid rotifers exposed to air (*T. lignicola* and *T. trigonosporum*). Their ascospores/conidia and mycelia survive in soil, or on various humus, rotting wood, plant tissues and surfaces, body surfaces of insects and mites, tissues of *Cordyceps* and lichens (Table 1).

*Species diversity and distribution*: *Tolypocladium* currently consists of 42 species (including the novel species described in this study) distributed worldwide [2,3,17]. Seventeen species were recorded from China (Table 1).

### 2.3. Description of the Novel Species

**Figure 2:**
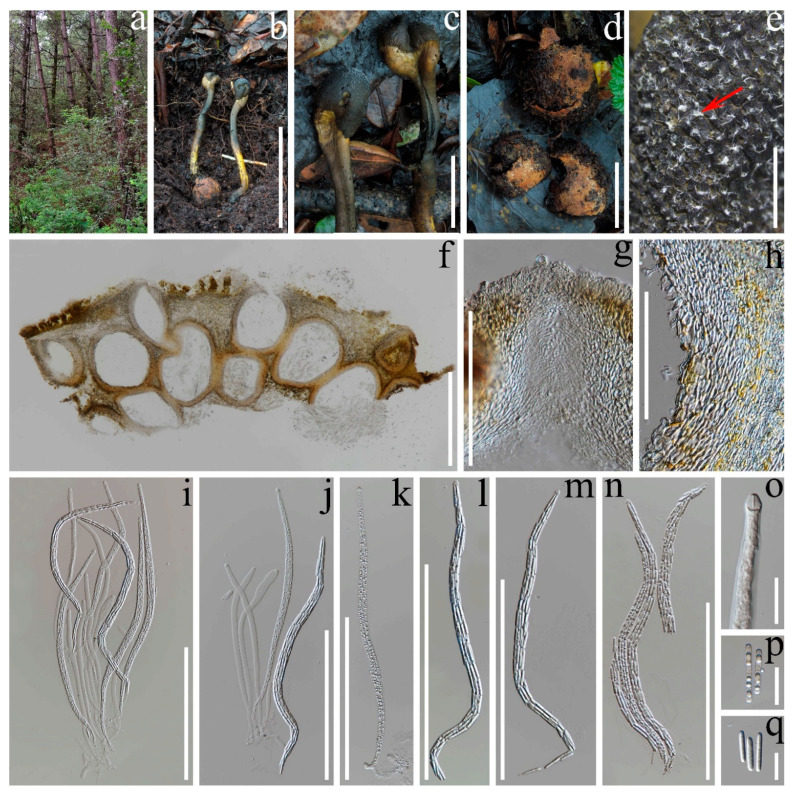
*Tolypocladium inusitaticapitatum* (holotype: HKAS 112152). (**a**) Habitat; (**b**) Stromata arising from the fruiting bodies of *Elaphomyces* sp.; (**c**) Fertile heads; (**d**) Decomposed *Elaphomyces* sp.; (**e**) Ascospores released from mature perithecia (shown by a red arrow); (**f**) Vertical section of a fertile head; (**g**) Median section across the ostiole of the perithecium; (**h**) Vertical section across the cortex of a fertile head; (**i**–**n**) Asci with ascospores; (**o**) A thickened cap; (**p**,**q**) Part-spores. Bars: (**b**) = 10 cm; (**c**,**d**) = 2 cm; (**e**) = 2 mm; (**f**) = 500 μm; (**g**) = 50 μm; (**h**) = 100 μm; (**i**–**n**) = 250 μm; (**o**–**q**) = 20 μm.

*Tolypocladium inusitaticapitatum* F.M. Yu, Q. Zhao and K.D. Hyde, sp. nov. Figure 2.

Index Fungorum: IF558123; Facesoffungi number: FoF 10407.

*Typification*: China, Yunnan Province, Lijiang City, Lijiang Alpine Botanic Garden, E100°10′58.07, N26°59′58.35, alt. 3338 m, 5 Oct 2019, Jian-Wei Liu (HKAS 112152, holotype).

*Etymology*: The specific epithet ‘inusitaticapitatum’ is derived from the combination of two Latin words, 1) adjective inusitata (strange, odd) and 2) noun capitatum (head), pointing to the fertile head, which is irregularly expanded.

*GenBank accession numbers*: ITS = MW 537735, LSU = MW 537718, SSU = MW 537733, *TEF*1-*α* = MW 507527, *RPB*2 = MW 507529.

*Description*: Asexual morph *Stromata* 9–11.5 cm high, solitary and simple, arising directly from the fruiting bodies of *Elaphomyces* sp. *Stipe* yellow at base, olive-brown to dark brown at the middle part, and yellowish brown at the terminal part. They are 7.5–11.5 cm long and 7–8.5 mm thick in the widest parts and nearly cylindrical, but the middle part is slightly thicker than the basal and upper parts. The *fertile part* developed from the terminal of the stipe, and is somewhat ellipsoidal, irregularly barrel-shaped, and sometimes slightly compressed, 1.5–2.0 cm × 1.5–2.0 cm. The surface is decorated with white ascospores released from the mature perithecia, which is olive yellow when immature, and olive to dark brown when mature. The outer layer becomes cracked and the olive internal texture is exposed. *Structure of cortex of fertile part:* composed of olive brown pseudoparenchymatous tissue and an ectal layer. *Perithecia* 580–720 μm × 180–270 μm (x = 650 μm × 220 μm, *n* = 10), crowded, entirely immersed, obovoid, ellipsoidal to pyriform. *Ostioles* papillate, and are visible (protruding up to 55 μm in high) or invisible, lined with periphyses. *Asci* is 410–510 μm × 10–15 μm (x = 461 μm × 13 μm, *n* = 20), hyaline, and long cylindrical, with a conspicuously thickened cap (measuring 6.5–7.5 μm × 6.0–7.0 μm). *Ascospores* are approximately as long as asci, and extremely easy to break into part-spores. *Part-spores* 20–32 μm × 3.0–4.5 μm (x = 25 μm × 3.6 μm, *n* = 20), hyaline, cylindrical with rounded ends. Asexual morph: Unknown.

*Host and habitat*: Directly arising from the fruiting bodies of hypogeous *Elaphomyces* sp. (*Elaphomycetaceae*, *Eurotiales*), in a humid and evergreen broad-leaved rainforest (Lijiang Alpine Botanic Garden), Lijiang, Yunnan Province, P.R. China. As serious degradation has occurred, truffle-like *Elaphomyces* sp. could not show any morphological evidence of taxonomic significance. Based on the ITS sequence dataset, the phylogenetic analyses showed that the host of *T. inusitaticapitatum* clustered together with *Elaphomyces fuscus* M. Shirakawa (Japan) and formed a sister group. However, there are sufficient molecular differences between the host from HKAS 112152 (ITS = MW 513695) and *E. fuscus* F-a170629 (ITS = LC 500967) to consider them as distinct species.

*Known distribution*: P.R. China (Yunnan).

*Other specimen examined*: CHINA, Yunnan, Lijiang, Lijiang Alpine Botanic Garden, alt. 3338 m, 5 October 2019, Jian-Wei Liu (HKAS 112153).

*Notes*: Based on the multi-gene phylogeny results, our specimens are closely related to *Tolypocladium flavonigrum,* known only from Thailand. Both species have stromata directly emerging from the surface of *Elaphomyces* sp., and capitate fertile heads with the perithecia entirely immersed in a well-differentiated valliforme-like structure [30]. However, *T. inusitaticapitatum* considerably differs from *T. flavonigrum* for the olive, yellowish-brown to dark brown fertile part, and is yellow to yellowish-brown at both ends of the stipe compared to the yellow–black to black stromata in *T. flavonigrum*. *Tolypocladium inusitaticapitatum* produces obovoid, ellipsoidal to pyriform perithecia, which are markedly distinguished from the elongate-ovoid perithecia produced by *T. flavonigrum*. Asci and part-spores of *T. inusitaticapitatum* (410–510 μm × 10–15 μm, 20–32 μm × 3.0–4.5 μm) are larger than those of *T. flavonigrum* ((318–)330–416(–482) μm × 7–8 μm, 2–5 μm × 1.5–2 μm) [30].

When comparing *Tolypocladium inusitaticapitatum* with its other phylogenetic relatives (*T. capitatum*, *T. fractum* and *T. longisegmentatum*), differences were found. *Tolypocladium capitatum* differs from *T. inusitaticapitatum* mainly due to its larger perithecia (900–1100 μm × 340–430 μm) and slimmer part-spores (2.5–3 μm wide) [10]. *Tolypocladium fractum* differs from *T. inusitaticapitatum* by having smaller stromata (1.5–2.5 cm long) and asci (300–480 μm × 5–6 μm) [10]. *Tolypocladium longisegmentatum* is distinguished from *T. inusitaticapitatum* by its longer stipe (13 cm long when fresh and up to 11 cm long when dried) and longer part-spores ((12–)40–65 μm) [20]. Morphologically, *T. inusitaticapitatum* is similar to *T. intermedium* for the yellow to dark brown stipe but differs in its smaller asci and shorter part-spores (main differences are outlined in Table 3). Regretfully, the molecular data of *T. intermedium* is not available in GenBank.

## 3. Discussion

*Tolypocladium*, a generalist genus, has been reported to have diverse lifestyles on a wide range of hosts and environments, including soil, insects, plants, lichens and hypogeal fungi [6,8]. The current pattern of host affiliation of *Tolypocladium* fungi is inferred to be an evolutionary product of intra- and inter-kingdom host shifts [57]. In the last two decades, researchers aimed to infer the evolution of host affiliation within the *Tolypocladium,* either using a handful of gene loci from dozens to hundreds of taxa, or using genome-scale data from fewer taxa [11,12,15,58]. To date, the studies on the host-jumping of *Tolypocladium* have been performed with multigene phylogeny (seven genes from 202 taxa of *Hypocreales*) [12] and genome-scale phylogeny (1350 genes from 20 taxa of *Hypocreales*) [15]. The multigene phylogenies supported three hypotheses for *Tolypocladium*, as follows: (1) the ancestral hosts were fungi (false truffles) [11,12,57,58]; (2) there were multiple switches to insect pathogenesis from a mycoparasitic ancestor [8,12,13]; (3) the endophytic lineage has arisen with the contact of plant hosts via mycorrhizal associations or plant-associated insects [12]. However, these conclusions, made from multigene phylogenies, conflict with those made from genome-scale phylogenies, which suggested a single ecological transition from insects to fungi within *Tolypocladium* [15]. Our phylogenic tree, inferred from five genes of 35 species (Figure 1), resulted in consistent conclusions, similar to those from previous multigene phylogenies. Similarly, we encountered several problems, such as phylogenetic conflicts among genetic data partitions and moderate to low support values for some important nodes [8,12,13]. Although whole-genome data provide insights that can further resolve the phylogenetic relationships of *Tolypocladium* [15,59,60], it is still unknown whether those conclusions will be limited by the few available species.

In this study, a novel *Tolypocladium* species occurring on *Elaphomyces* sp. is known from its sexual morph. A taxonomic key is also provided for 26 *Tolypocladium* species. The shape of the fertile part, the connection between the stipe and host, the structure of the cortex of the fertile part, size of part-spores and host affiliation are thought to be characteristic of taxonomic significance for interspecific identification [8,9,10]. However, there are 16 species whose sexual morphs are still unknown. In addition, the phylogenetic relationships among *Tolypocladium* species are very sensitive to taxa sampling and loci information [8,15]. Further studies should focus on obtaining more samples from different geographic regions and/or ecological niches, sequencing more markers and even genomic data, building a more robust phylogenetic relationship, and establishing their sexual–asexual morph connections. (Table 4).

## 4. Materials and Methods

### 4.1. Collections and Morphology

*Tolypocladium* specimens, including their underground host *Elaphomyces* sp., were collected in an evergreen broad-leaved forest in Lijiang Alpine Botanic Garden, Lijiang City, Yunnan Province, China. The specimens were examined as described in Senanayake and colleagues with the following modifications [61]. Colour codes were recorded following those of Kornerup and Wanscher [62]. Specimens were deposited at the Herbarium of Cryptogams Kunming Institute of Botany, Chinese Academy of Sciences, Kunming, China (HKAS, KUN).

### 4.2. DNA Extraction, PCR Amplification and Sequencing

The genomic DNA was extracted from the dried materials following the method described by Dissanayake and colleagues [63]. Fertile tissues from the parasitic fungi and the peridium of the host mushroom were used to extract DNA. Primer pairs ITS1F/ITS4 [64], LR0R/LR5 [65,66], PNS1/NS8 [64], *TEF*1-*α* 983F/*TEF*1-*α* 2218R [67] and f*RPB*2-5F/f*RPB*2-7R [68] were used for the amplification of the internal transcribed spacer region ITS1-5.8S-ITS2 (ITS), the large subunit rDNA (LSU), the small subunit rDNA (SSU), the translation elongation factor 1-*α* (*TEF*1-*α*) gene and RNA polymerase II second-largest subunit (*RPB*2), respectively. PCR reaction was performed in a 25 μL reaction volume, comprising 12.5 μL Taq PCR Master Mix (Abmgood, Richmond, BC, Canada), 1 μL forward primer, 1 μL reverse primer, 2 μL DNA template and 8.5 μL ddH2O. For ITS, LSU, SSU and *RPB*2, PCR reaction conditions were as follows: 5 min at 94 °C, followed by 35 cycles of 40 s at 94 °C, 40 s at 53 °C and 1 min at 72 °C, and a final extension of 10 min at 72 °C. PCR reaction condition of *TEF*1-*α* was as follows: 5 min at 94 °C, followed by 35 cycles of 50 s at 94 °C, 40 s at 64 °C and 1 min at 72 °C, and a final extension of 10 min at 72 °C. The PCR products were visualized using agarose gel electrophoresis after staining with dyes (TS-GelRed Ver.2, Tsingke Biotechnology Co., Ltd., Beijing, China). Then, the products were sent for sequencing at Sangon Biotech Co. Ltd., Shanghai, China.

### 4.3. Sequence Alignment and Phylogenetic Analyses

Phylogenetic trees were constructed using the sequencing data of *T. inusitaticapitatum* and the allied reference sequences of closely related *Ophiocordycipitaceae* species obtained from the GenBank (Table 5). *Aschersonia confluens* (BCC 7961) and *A. paraphysata* (BCC 1467) of *Clavicipitaceae* were used as outgroup taxa. All sequences were assembled and aligned using MAFFT v 6.8 [69] and manually edited where necessary in BioEdit version 7.0.9 [70]. Individual alignments were compiled for SSU, LSU, ITS, *TEF*1-*α* and *RPB*2 genes. The optimal substitution model for each gene dataset was determined using MrModeltest 2.3 [71] under the Akaike information criterion (AIC). The results indicated that the GTR+I+G model was optimal for all the gene regions. Individual datasets were combined to assemble the combined dataset (gene order: SSU, LSU, ITS, *TEF*1-*α* and *RPB*2). The resulted combined dataset was deposited in the TreeBASE database (http://purl.org/phylo/treebase/phylows/study/TB2:S27887?x-access-code=746eddc746009259527edd3d4c69526b&format=html, accessed on 10 March 2021).

Maximum likelihood (ML) analysis was performed using IQ-Tree (http://iqtree.cibiv.univie.ac.at/, accessed on 20 May 2021) [72,73]. The substitution model options for each gene were auto-evaluated according to the provided partition file. Clade support for the ML analysis was assessed using an SH-aLRT test with 1000 replicates [74] and the ultrafast bootstrap (UFB) [75]. In the ML analyses, nodes with support values of SH-aLRT ≥ 80 and UFB ≥ 95 were considered well-supported, those with either SH-aLRT < 80 or UFB < 95 were considered weakly supported, and nodes with SH-aLRT < 80 and UFB < 95 were considered unsupported.

Bayesian Inference (BI) analysis was carried out in MrBayes v3.2.6 [76]. Gaps were treated as missing data. Four simultaneous Markov Chain Monte Carlo (MCMC) chains were run for 10,000,000 generations and were sampled at every 100th generation until the standard deviation of the split frequencies fell below 0.01 and ESS values > 200. Subsequently, phylogenetic trees were summarized and posterior probabilities (PP) were calculated using MCMC by discarding the first 25% generations as the burn-in phase [77]. Phylogenetic trees were viewed in FigTree v.1.4.4. Nodes with BI posterior probability (BIPP) > 0.90 were considered to be well supported.

## Figures and Tables

**Table 1:** Species diversity, hosts/habitats and geographic distribution of *Tolypocladium* species.

Fungal Name	Hosts/Isolated From	Known Distribution
*T. album*	Soil, sapwood of *Hevea brasiliensis*	Colombia, France, Scotland, Sri Lanka, Sweden, The Netherlands [7], Peru [12]
*T. amazonense*	Sapwood of *Hevea brasiliensis* and *H. guianensis*	Peru [12]
* T. capitatum	*Elaphomyces granulatus*, *E. japonicus*, *Elaphomyces* sp.	Asia (China (Taiwan, Yunnan), Japan), Europe (France, Holland, Hungary), North America (Canada, U.S.A.) [9,10,19,20,21,22]
*T. cylindrosporum*	Soil, sewage, peat, roots of *Picea mariana*; *Plecia nearctica*, larvae of *Aedes sierrensis*, larvae of *Aedes australis*, larvae and pupae of *Lucilia sericata*, *Drosophila* larvae (*Diptera*)	Brazil, China, Czech, England, New Zealand, Nepal, The Netherlands, The North Island, U.S.A. [4,23,24,25,26,27]
* T. delicatistipitatum	*E. asahimontanus*	China (Jiangxi) [28], Japan [10]
* T. dujiaolongae	Cicada nymphs	China (Anhui, Fujian, Jiangsu, Jiangxi, Zhejiang) [29]
*T. endophyticum*	Living sapwood of *Hevea brasiliensis* and *H. guianensis*	Brazil, Mexico, Peru [12]
*T. extinguens*	Larvae of *Arachnocampa luminosa* (*Diptera*)	New Zealand [24]
* T. fractum	*E. appalachiensis*	U.S.A. (Tennessee) [9]
* T. flavonigrum	*Elaphomyces* sp.	Thailand [30]
* T. fumosum	Cocooned pupa of bagworm moth (*Psychidae*) buried among mosses	Poland [31]
*T. geodes*	Soil	Austria, Canada, China, Denmark, England, The Netherlands [4,23,26]
* T. guangdongense	*Elaphomyces* sp.	China (Guangdong) [32]
* T. inegoense	Cicada nymphs (e.g., *Hyalessa maculaticollis*)	China (Fujian, Taiwan) [33], Japan [34], Korea [6]
* T. inflatum	Larvae of *Scarabaeidae* (e.g., *Aphodiinae*, *Rutelinae*) (sexual morph); soil, humus, *Picea glauca*, roots of *P. mariana*, surface of *Mycobates* sp. (*Acari*, *Mycobatidae*), sclerotium of *Ophiocordyceps gracilis* (asexual morph)	Sexual morph: Japan, U.S.A. (Tennessee, North Carolina, Michigan, New York, Washington) [5]; asexual morph: Austria, Canada, China, Nepal, Germany, U.S.A. [4,23,26,35]
* T. intermedium	*E. granulatus*, *E. subvariegatus*	Japan, U.S.A. (New York) [10,36]
* T. japonicum	*E. granulatus*, *E. japonicus*, *E. neoasperulus*	Austria, Japan [10], China (Guizhou, Taiwan) [28,37]
* T. jezoense	*E. anthracinus*, *E. miyabeanus*, *E. nopporensis*	Japan [10]
*T. lignicola*	Rotting wood (parasitic in bdelloid rotifers)	Canada (Ontario) [38]
* T. longisegmentatum	*E. granulatus*, *E. japonicus*, *E. muricatus*, *Elaphomyces* sp.	Asia (China (Jilin), Japan), Europe (England, Germany, Holland), North America (Canada, Mexico, U.S.A.) [9,10,20,21,39]
*T. microsporum*	Soil	Canada, Germany, The Netherlands, U.S.A. [23]
* T. minazukiense	*Elaphomyces* sp.	Japan [40]
* T. miomoteanum	*Elaphomyces* sp.	Japan [40]
*T. nubicola*	Soil	Canada (Alberta), China (Guizhou) [23,41]
* T. ophioglossoides	*E. granulatus*, *E. japonicus*, *E. muricatus*, *E. shimizuensis*, *E. titibuensis*, and *Elaphomyces* sp.	Commonly in Asia (e.g., China (Guangxi, Jiangsu, Jiangxi, Jilin, Shandong, Sichuan, Taiwan, Yunnan), Japan, Korea), Europe and North America [9,10,42,43,44]
*T. ovalisporum*	Lichen *Polycauliona regalis*	Antarctica (King George Island) [45]
* T. paradoxum	Cicada nymphs (e.g., *Platypleura kaempferi*, *Graptopsaltria nigrofuscata*)	China (Hainan, Yunnan) [46], Japan, Koera [34,47]
*T. pustulatum*	Soil, twigs in oak forest, and living leaf of *Kalmia latifolia*	Mexico (Nuevo León), Spain (Cádiz), U.S.A. (New Jersey) [48]
* T. ramosum	*Elaphomyces* sp.	China (Anhui, Fujian, Gansu, Guangdong) [44,49,50]
* T. rouxii	*E. variegatus*	France [51]
*T. sinense*	Stroma and sclerotium of *Ophiocordyceps sinensis*	China (Yunnan) [52]
* T. szemaoense	*E. granulatus*	China (Yunnan) [53]
* T. tenuisporum	Host not found (probably *Elaphomyces* sp.)	U.S.A. (Pennsylvania) [9]
*T. terricola*	Soil	Finland [54]
* T. toriharamontanum	Cicada nymph (*Auritibicen bihamatus*)	Japan [34]
*T. trigonosporum*	Rotting stump (parasitic on bdelloid rotifers)	Canada (Nova Scotia) [55]
*T. tropicale*	Sapwood and leaf tissue of *Hevea brasiliensis*	Mexico, Peru [12]
*T. tundrense*	Soil	Canada (Northwest Territories) [23]
* T. valliforme	*E. granulatus*, *Elaphomyces* sp.	Canada (Ontario), U.S.A. (Carolina, New York, Virginia) [9]
* T. valvatistipitatum	*E. granulatus*, *E. neoasperulus*	Japan [10]
* T. virens	*Elaphomyces* sp.	Japan [56]

* indicates sexual morphs (25 species).

**Table 2:** Intraspecific base-pair differences in LSU genes among *Tolypocladium* species.

	Locus	522	532	855									Ratio
Species													
*T. album*	CBS 393.89 ^#^	C	C	C									0.35% (3/870 bp)
	GB5502	T	T	-									
	Locus	20	21	23	24	25	27						Ratio
Species													
*T. inflatum*	OSC 71235 ^#^	A	G	A	A	C	A						0.76% (6/794 bp)
	CBS 127302	G	A	-	-	-	C						
	Locus	48	434										Ratio
Species													
*T. ophioglossoides*	CBS 100239 ^#^	C	C										0.25% (2/816 bp)
	NBRC 106330	T	T										
	Locus	164	382	405	433	442	479	496	524				Ratio
Species													
*T. paradoxum*	NBRC 106958 ^#^	T	C	G	C	C	C	T	G				0.90% (8/891 bp)
	NBRC 100945	C	T	A	T	T	T	C	A				
	Locus	8	37	44	51	81	96	110	124	204	210	402	Ratio
Species													
*T. inusitaticapitatum*	HKAS 112152 ^#^	T	T	A	A	A	A	T	T	A	A	G	1.28% (11/862 bp)
	HKAS 112153	C	C	G	G	G	G	C	C	G	G	T	

The locus numbers refer to the base-pair positions of the gene sequences, and “^#^” represents the reference sequences. Gaps are indicated with ‘-’.

**Table 3:** Main differences between *T. intermedium* and *T. inusitaticapitatum*.

	*T. intermedium* [10]	*T. inusitaticapitatum* (This Study)
Fertile part	Dark reddish brown	Olive brown, yellowish-brown to dark brown
Stipe	Slender, 6–8.5 cm long and 2–4 mm thick, middle part clearly expanded, surface with many longitudinal grooves, upper part squamulose	Thicker, 7.5–11.5 cm long and 7–8.5 mm thick, middle part indistinctly expanded, surface smooth
Asci	240–300 μm × 7–8 μm, caps about 5 μm in diameter	410–510 μm × 10–15 μm, caps 6.5–7.5 μm × 6.2–7.0 μm
Part-spores	Short, 3–6 (commonly 4.5) μm × 1.5–2 μm, truncated at two ends (shape)	Long, 20–32 μm × 3.0–4.5 μm, cylindrical with rounded ends
Distribution	Japan, USA	P.R. China (Yunnan)

**Table 4:** Key to Sexual Morphs of *Tolypocladium* species.

1. Host insects	2
1′. Host hypogeous *Elaphomyces* spp.	7
2. Host beetle or moth larvae	3
2′. Host cicada nymphs	4
3. Fertile part capitate, with stellate appearance; perithecia ovoid to pear-shaped, 740–760 × 444–558 μm	*T. fumosum*
3′. Fertile part, strap-shaped pseudostalk; perithecia superficial, narrow flask-shaped, 1000–1500 × 330–440 μm	*T. inflatum*
4. Stromata arising from underground mycelial membrane or strand; part-spores 3–5 × 1.5–2 μm	*T. paradoxum*
4′. Stromata arising directly from host	5
5. Fertile part elongated, obpyriform; part-spores 1.5–2–2.5 × 1.5–1.7 μm wide	*T. toriharamontanum*
5′. Fertile part oblong or clavate	6
6. Perithecia superficial or apparently half-immersed, pyriform, 520–550 × 260–280 μm; part-spores 2.5–3 × 2 μm	*T. inegoense*
6′. Perithecia wholly immersed, ampullaceous, (233–)520–740(–780) × (250–)300–330 (–360) μm; part-spores 3–5(–7.0) × 2–3 μm	*T. dujiaolongae*
7. Stroma attached to host by rhizomorphs	8
7′. Stroma arising directly from the host	12
8. Part-spores articulate, moniliform, 3–3.5 × 2–2.5 μm	*T. szemaoense*
8′. Part-spores with truncate or rounded ends	9
9. Stroma capitate	10
9′. Stroma solitary or rarely caespitose	11
10. Perithecia small, 480–540 × 225–255 μm; part-spores large-sized, 18–28 × 3–5 μm	*T. delicatistipitatum*
10′. Perithecia 770–800 × 350–430 μm; part-spores medium-sized, 8–11 × 1.5–2 μm	*T. miomoteanum*
11. Perithecia oblong with long neck, 700–720 × 200–250 μm; part-spores long, 20–30(50) × 3–4.5 μm	*T. jezoense*
11′. Perithecia ovoid, 550–600 × 200–300 μm; part-spores small short rod-shaped, 2.5–5 × 1.5–2 μm	*T. ophioglossoides*
12. Perithecia superficial, ascospores nonfractured	*T. ramosum*
12′. Perithecia entirely embedded or ostiole slightly projecting	13
13. Fertile part, cortex composed of pseudoparenchymatous peridial layer, and with an ectal layer	14
13′. Fertile part, cortex composed of pseudoparenchymatous peridial layer, but without ectal layer	19
14. Stromata clavate; perithecia narrowly ovoid, 750–1000 × 250–300 μm; part-spores cylindric, 6–8 × 1–1.5 μm	*T. tenuisporum*
14′. Stromata capitate	15
15. Part-spores, larger-sized, more than 20 μm long	16
15′. Part-spores, less than 20 μm long	17
16. Part-spores (12–)40–65 × (3–)4–5 μm	*T. longisegmentatum*
16′. Part-spores 20–32 × 3.0–4.5 μm	*T. inusitaticapitatum*
17. Part-spores, medium-sized, (13–)16(–21) × 2.5–3 μm	*T. rouxii*
17′. Part-spores, small-sized, 2.5–6 μm long	18
18. Perithecia elongate-ovoid, (560–)567–697(–750) × (200–)206–248(–250) μm; part-spores 2–5 × 1.5–2 μm	*T. flavonigrum*
18′. Perithecia ovoid, 450–540 μm × 230–260 μm; part-spores 3–6 (commonly 4.5) × 1.5–2 μm	*T. intermedium*
19. Stromata clavate	20
19′. Stromata capitate	21
20. Perithecia small, 245–495 μm long, deeply embedded; asci short, 195–270 μm long	*T. guangdongense*
20′. Perithecia 500–550 μm long, ostiola slightly projecting; asci 330–370 μm long	*T. japonicum*
21. Perithecia large, more than 900 μm long	22
21′. Perithecia medium-sized, 400–700 μm long	23
22. Perithecia ovoid, 900–1100 × 340–430 μm; part-spores cylindric or somewhat fusoid, 18–27 (commonly 24) × 2.5–3 μm	*T. capitatum*
22′. Perithecia ampullaceous, 900–930 × 220–250 μm; part-spores fusoid, 16–18 × 3 μm	*T. minazukiense*
23. Stipe slender, less than 1.0 mm thick	24
23′. Stipe thick, columnar, 1.0–6.0 mm thick	25
24. Perithecia 500–600 × 250–350 μm; part-spores 2–5 × 1.5–2 μm	*T. fractum*
24′. Perithecia 400 × 250 μm; part-spores 6 × 1.5 μm	*T. virens*
25. Asci 10–12 μm wide; part-spores medium-sized, 7.5–16 × 2.5–3 μm	*T. valvatistipitatum*
25′. Asci slender, 6–8 μm wide; part-spores small-sized, 3–8 × 2 μm	*T. valliforme*

**Table 5:** Voucher information and GenBank accession numbers for samples appearing in the *Tolypocladium* phylogenetic tree.

Taxon	Strain/Specimen Voucher	GenBank Accession Numbers				
		ITS	28S	18S	*TEF*1-*α*	*RPB*2
*Aschersonia confluens*	BCC 7961	JN049841	DQ384947	DQ372100	DQ384976	DQ452465
*A. paraphysata*	BCC 1467		DQ377987	DQ372090	DQ384967	DQ452463
*Drechmeria gunnii*	OSC 76404	JN049822	AF339522	AF339572	AY489616	DQ522426
*D. sinensis*	CBS 567.95	MH862540	AF339545	AF339594	DQ522343	DQ522443
***D. zeospora* ^ex^**	**CBS 335.80**	**MH861269**	**AF339540**	**AF339589**	**EF469062**	**EF469109**
*Ophiocordyceps gracilis*	EFCC 8572	JN049851	EF468811	EF468956	EF468751	EF468912
*O. heteropoda*	EFCC 10125	JN049852	EF468812	EF468957	EF468752	EF468914
*Paecilomyces lilacinus*	CBS 431.87	AY624188	EF468844		EF468791	EF468940
***Pa. lilacinus* ^ex^**	**CBS 284.36**	**AY624189**	**FR775484**		**EF468792**	**EF468941**
*Perennicordyceps cuboidea*	NBRC 101740	JN943331	JN941417	JN941724	KF049684	
*Pe. cuboidea*	NBRC 100941	JN943329	JN941416	JN941725		
*Pe. paracuboidea*	NBRC 101742	JN943338	JN941431	JN941710	KF049685	KF049669
*Pe. paracuboidea*	NBRC 100942	JN943337	JN941430	JN941711	AB972954	AB972958
*Pe. prolifica*	TNS-F-18481	KF049659	KF049631	KF049612	KF049686	
*Pe. prolifica*	TNS-F-18547	KF049660	KF049632	KF049613	KF049687	KF049670
***Polycephalomyces aurantiacus***	**MFLU 17-1393**	**MG136919**	**MG136913**	**MG136907**	**MG136877**	**MG136873**
*Po. aurantiacus*	MFLUCC 17 2113	MG136916	MG136910	MG136904	MG136875	MG136870
***Po. marginaliradians***	**MFLU 17-1582**	**MG136920**	**MG136914**	**MG136908**	**MG136878**	**MG271931**
*Po. marginaliradians*	MFLUCC 17-2276	MG1369 21	MG136915	MG136909	MG136879	MG271930
*Po. nipponicus*	NBRC 101406	JN943301	JN941388	JN941753		
*Po. nipponicus*	BCC 1682	KF049664	KF049638	KF049620	KF049694	MF416463
*Po. yunnanensis*	YHCPY1005	KF977848	KF977848	KF977848	KF977850	KF977854
***Po. yunnanensis***	**YHHPY1006**	**KF977849**	**KF977849**	**KF977849**	**KF977851**	**KF977855**
*Tolypocladium amazonense*	VPB179	KF747267		KF747329		
***T. amazonense* ^ex^**	**MS308**		**KF747134**	**KF747314**	**KF747099**	
*T. capitatum*	NBRC 106325		JN941402	JN941739	AB968598	AB968559
*T. capitatum*	NBRC 100997		JN941401	JN941740	AB968597	AB968558
***T. cylindrosporum***	**ARSEF 2920**	**MG228381**			**MG228390**	**MG228387**
*T. cylindrosporum*	YFCC 1805001	MK984581	MK984577	MK984565	MK984569	MK984573
*T. endophyticum*	MX535	KF747260	KF747153	KF747322	KF747117	
***T. flavonigrum* ^ex^**	**BCC 66576**	**MN338090**	**MN337287**		**MN338495**	
*T. flavonigrum*	BCC 66578	MN338091	MN337288		MN338496	
*T. flavonigrum*	BCC66580		MN337289		MN338497	
*T. fractum*	OSC 110990		DQ518759	DQ522545	DQ522328	DQ522425
***T. fumosum***	**WA18945**	**KU925171**	**KU985053**			
*T. geodes*	CBS 126054	MH864065	MH875520			
*T. inflatum*	OSC 71235	JN049844	EF469077	EF469124	EF469061	EF469108
*T. inflatum*	CBS 127302	MH864514	MH875949			
***T. inusitaticapitatum***	**HKAS 112152**	**MW537735**	**MW537718**	**MW537733**	**MW507527**	**MW507529**
*T. inusitaticapitatum*	HKAS 112153	MW537736	MW537719	MW537734	MW507528	MW507530
*T. jezoense*	txid94205	AB027365	AB027365	AB027319		
*T. longisegmentatum*	OSC 110992		EF468816			EF468919
***T. nubicola***	**CBS 568.84**	**MH861780**	**MH873478**			
*T. ophioglossoides*	CBS 100239	KU382155	KJ878874	KJ878910	KJ878958	
*T. ophioglossoides*	NBRC 8992	JN943316	JN941405	JN941736	AB968601	AB968562
***T. ovalisporum***	**CBS 700.92**	**AB457006**				
*T. paradoxum*	NBRC 106958	JN943324	JN941411	JN941730	AB968600	AB968561
*T. paradoxum*	NBRC 100945	JN943323	JN941410	JN941731	AB968599	AB968560
*T. pustulatum*	MRL GB6597	AF389189	AF389190			
*T. tropicale*	MX338	KF747259	KF747149	KF747318	KF747113	
***T. tropicale* ^ex^**	**IQ214**	**KF747254**	**KF747125**		**KF747090**	
***T. tundrense***	**CBS 569.84**	**MH861781**	**MH873479**			
*T. valliforme*	DAOM 196368	AY245640		AY245648		

New sequencing data are displayed in bold. Specimens of the current study are given in red. Type specimens are in bold; superscript ‘ex’ indicates ex-type.

## Data Availability

The obtained gene sequences were deposited in the NCBI GenBank database. The accession numbers of the obtained sequences are contained within the article and in Table 5.
